# T2-weighted cardiovascular magnetic resonance in acute cardiac disease

**DOI:** 10.1186/1532-429X-13-13

**Published:** 2011-02-18

**Authors:** Ingo Eitel, Matthias G Friedrich

**Affiliations:** 1 University of Leipzig - Heart Center, Department of Internal Medicine - Cardiology, Leipzig, Germany; 2 Stephenson Cardiovascular Magnetic Resonance Centre at the Libin Cardiovascular Institute of Alberta, Departments of Cardiac Sciences and Radiology, University of Calgary, Calgary, Canada

## Abstract

Cardiovascular magnetic resonance (CMR) using T2-weighted sequences can visualize myocardial edema. When compared to previous protocols, newer pulse sequences with substantially improved image quality have increased its clinical utility. The assessment of myocardial edema provides useful incremental diagnostic and prognostic information in a variety of clinical settings associated with acute myocardial injury. In patients with acute chest pain, T2-weighted CMR is able to identify acute or recent myocardial ischemic injury and has been employed to distinguish acute coronary syndrome (ACS) from non-ACS as well as acute from chronic myocardial infarction.

T2-weighted CMR can also be used to determine the area at risk in reperfused and non-reperfused infarction. When combined with contrast-enhanced imaging, the salvaged area and thus the success of early coronary revascularization can be quantified. Strong evidence for the prognostic value of myocardial salvage has enabled its use as a primary endpoint in clinical trials. The present article reviews the current evidence and clinical applications for T2-weighted CMR in acute cardiac disease and gives an outlook on future developments.

"The principle of all things is water"

Thales of Miletus (624 BC - 546 BC)

## Introduction

Cardiovascular magnetic resonance (CMR) is well-established and increasingly used in clinical practice for the diagnosis and management of cardiovascular disease [1-3].

Importantly, recent technological advances of CMR have introduced its use for visualizing certain tissue changes in patients with acute myocardial diseases. This is of particular interest in patients with suspected ischemic disease, a broad and heterogeneous population that challenges the clinician in terms of: 1) accurately establishing the diagnosis; 2) risk stratification; 3) therapeutic decision making; and 4) monitoring response to therapy [4].

CMR is uniquely able to integrate, in a single examination, an accurate quantitative assessment of left ventricular (LV) function, structural abnormalities of the myocardial tissue including edema, infarct size, and myocardial salvage as well as its microvascular status. Therefore, CMR has an unparalleled potential as the main diagnostic tool in acute cardiac disease by providing information on the stage, degree, and extent of reversible and irreversible myocardial injury [5,6].

Specifically, T2-weighted CMR has recently generated significant interest and has been employed to distinguish acute coronary syndrome (ACS) from non-ACS and recent from remote infarction in patients with undifferentiated chest pain [7-9]. Furthermore, T2-weighted CMR can be used to determine the area at risk in reperfused and non-reperfused myocardial infarction [10,11]. When combined with contrast-enhanced imaging of irreversible injury ("late gadolinium enhancement", LGE), the salvaged area at risk can be quantified and thus the success of early revascularization therapy can be assessed [12]. Moreover, myocardial salvage assessment has been shown to be independently associated with adverse cardiac events, opening new perspectives on its use as primary endpoint in clinical trails and in studies testing novel reperfusion strategies [13].

Edema imaging is also useful in other acute cardiac diseases, such as transplant rejection [14,15], myocarditis [16,17], as well as stress (Takotsubo) cardiomyopathy [18-20] and the clinical role continues to expand. Therefore, it is timely to review T2-weighted CMR, state-of-the-art techniques, limitations and its clinical usefulness for acute cardiac disease. We will discuss the level of evidence and give an outlook on future developments.

## Myocardial Edema

### Definition and Clinical Consequences

Edema (from 'óidema', the Greek word for 'swelling') is an elementary generic component of the tissue response to any acute injury regardless of its etiology (e.g. mechanic, toxic, ischemic) and therefore represents an important diagnostic target for assessing the acuity of tissue damage *in vivo *[6]. The term myocardial edema refers to both myocyte swelling (cytogenic edema) and fluid accumulation in the interstitial space (vasogenic edema). Several acute and chronic active conditions including myocardial infarction [21,22], reperfusion injury [23,24], inflammation [16,17], pulmonary hypertension [25], cardiopulmonary bypass [26-28], cardioplegic arrest [27], cardiac transplantation [29] and cardiac transplant rejection [14,15] are accompanied by myocardial edema.

**Figure 1:**
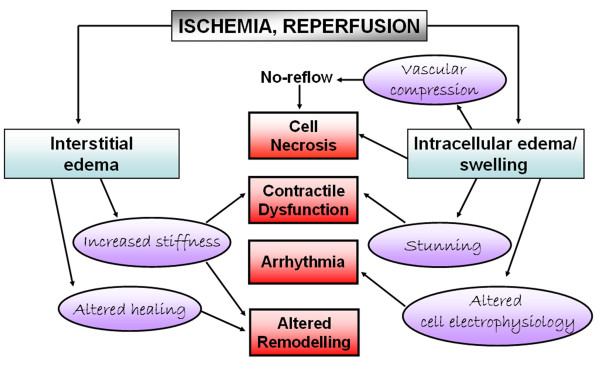
**Possible effects of myocardial edema induced by ischemia and reperfusion on myocardial function and survival**. Adapted with permission obtained from the Oxford University Press ^© ^Garcia-Dorado et al. Cardiovasc Res 1993, 27: 1555-63.

Of note, myocardial edema is not only a nonspecific yet invariable pathological concomitant of acute injury; it has also significant and relevant pathophysiological consequences itself. The presence of myocardial edema increases the stiffness and decreases the compliance of the LV [30,31]. In addition, a mere 3.5% increase in myocardial water content has been reported to result in the reduction of the cardiac output by 40% [32]. Increased hydrostatic pressure within the interstitial space can exacerbate the extent of necrosis by capillary compression [33]. Furthermore, edema may contribute to postischemic myocardial dysfunction (stunning), arrhythmia [24], and reduced ventricular compliance [34]. When chronic, myocardial edema results in further alteration of myocardial structure, most importantly in the development of myocardial fibrosis [35]. It is however not fully understood, how edema affects systolic and diastolic function, long-term tissue composition, and electrical stability. Figure 1 illustrates possible effects of edema caused by ischemia/reperfusion injury on myocardial function and myocyte injury/survival [24].

### Myocardial Edema Formation in Ischemia and Reperfusion

Almost 80% of the myocardium is water, of which 77% is held in intracellular components, whereas 23% are found in the intravascular and a very small in the interstitial compartment.

**Figure 2:**
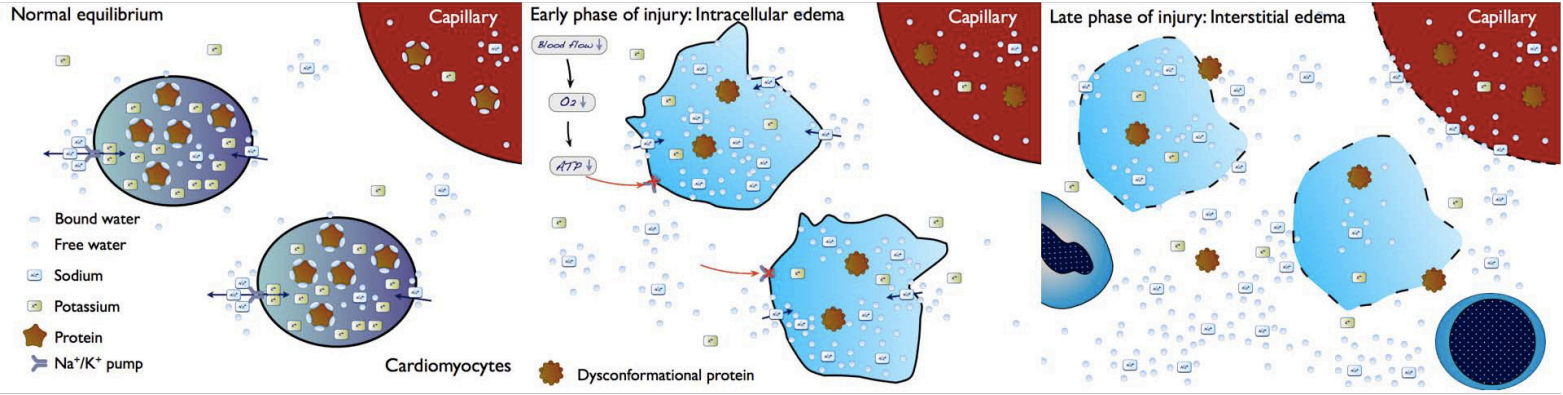
**A simplified schematic view of edema evolution in ischemic injury to the myocardium**. **A**: Normal equilibrium. **B**: Early phase of injury: intracellular edema. **C**: Late phase of injury: interstitial edema. Adapted with permission obtained from the Nature Publishing Group ^© ^Friedrich MG. Nat Rev Cardiol 2010, 3: 385-7.

Water molecules can permeate cellular or vascular membranes but under normal conditions the intracellular-extracellular water balance is kept in equilibrium by an active, ATP-dependent Na+/K+ exchange and by the binding of water to complex molecules such as intracellular proteins (Figure 2A).

Ischemia with or without reperfusion causes alterations in these fluid balance mechanisms leading to the development of cell swelling, increased interstitial water accumulation or both.

Very early after the onset of ischemia/hypoxia, water is released from proteins because of lactate-induced acidosis, increasing the intracellular fraction of free, unbound water [36]. Furthermore, failure of the ATP-dependent Na+/K+ channels results in intracellular Na+ accumulation with subsequent raise of the intracellular osmotic pressure and, accordingly, cellular water influx (Figure 2B). At this stage of purely intracellular edema, capillary membranes, more resistant to ischemia, are still intact. With persisting (>60-90 min) ischemia, capillary membranes lose their integrity and become permeable to complex molecules including proteins and plasma cells. Subsequent leakage of water from the intravascular space into the interstitial space [37] leads to net water inflow and interstitial edema (Figure 2C). Finally, cardiomyocyte death (oncosis) is followed by necrosis, a cascade of mostly inflammatory reactions for removing dead cells and debris. Reperfusion at this late stage of ischemic injury with inflow of normo-osmotic blood into this hyperosmotic region further enhances extracellular water accumulation and edema [24].

### Duration of Myocardial Edema After an Ischemic Event

It is unclear about how long myocardial edema persists after an ischemic event. In dogs the myocardial water content, as measured by histopathological methods, was still significantly increased in the infarct zone after 3 weeks [38]. In another canine model, Aletras et al [10] observed edema 2 months after infarction. Pathology studies in humans have shown complete resorption of edema after acute infarction within 5 weeks [39]. After alcohol-induced infarction in patients with hypertrophic obstructive cardiomyopathy edema was present after 28 days in all patients, whereas it was not found after 3 months [40]. Other studies in acute reperfused ST-elevation myocardial infarction patients have shown prolonged postinfarction edema after 6 or, in some patients, even 12 months [41,42]. Interestingly, persistence of myocardial edema after infarction varied from one week to 12 months in these studies. Reasons for persistent myocardial edema, particularly long after an ischemic event, might be due to increased wall stress and/or residual/recurring ischemia within the infarcted region [24]. Furthermore, compromised drainage of the infarct region owing to vessel damage and reparative processes, which last for months after an ischemic event, could cause persistent myocardial edema [41]. However, also artifacts of the T2-weighted imaging methodology have to be considered as a potential cause of prolonged edema presence. Thus, the exact duration and determinants of edema persistence after an ischemic event and possible prognostic implications remains to be studied further.

## Edema Assessment

In the past, edema could not be used as a diagnostic target/tool, because even histological techniques failed to provide reliable qualitative or quantitative data on its presence, extent, and regional distribution [6]. Three modalities have been used for the in vivo visualization of myocardial edema: Echocardiography, computed tomography (CT), and CMR.

As a surrogate for an increased myocardial water content, echocardiography relies on an increase of LV mass and wall thickness. Since the increase in LV mass is not specific for myocardial edema, the use of echocardiography to identify myocardial edema is limited, although recent advances in high-frequency ultrasound imaging techniques may be helpful [31,43].

Powell et al. [44] published pilot data on using CT for detecting myocardial edema. Poor temporal resolution and motion artefacts have limited the use of CT in clinical settings to quantify myocardial edema. Recently, Mahnken et al. [45] reported the ability of dual-source CT to detect myocardial edema in good agreement with CMR in a porcine acute myocardial infarction model. Confirmative studies in other clinical settings, however, are still lacking.

Using the water-sensitive properties of T2-weighted CMR, visualization of myocardial edema *in vivo *is possible within a few breath holds, without using radiation or contrast agents [6,46,47].

### CMR for the Assessment of Edema

Recent advances in T2-weighted CMR could significantly alleviate previous problems caused by an inherently low signal-to-noise ratio (SNR) and inconsistent image quality of previously used protocols. Many centers have implemented T2-weighted CMR as part of their standard protocols and there is a strong body of evidence on its useful application in several clinical settings [6,46]. Therefore, CMR has an exceptional role in identifying and quantification of myocardial edema *in vivo*.

In most CMR sequences, tissue contrast is mainly determined by the relaxation properties of protons after radiofrequency pulses. The long T2 relaxation times of water-bound protons are used to generate a water-specific contrast when applying T2-weighted sequences resulting in a high signal intensity of edematous tissue. In 1983, Higgins et al. [48] showed for the first time a direct relation between T2 (spin-spin) relaxation time and myocardial water content in a canine model of acute myocardial infarction, resulting in a positive correlation of tissue water content to the signal intensity on T2-weighted images. Similar to the brain, significant T2 lengthening has been observed in acute ischemia, accompanying a 3-5% increase in overall water content [48]. Importantly, in reperfused infarcts the water content may increase by as much as 28% [24].

The absolute water content, however, is not the only mechanism with a strong impact on T2. As the T2 of free water is about 40 times longer than that of bound water [49], the T2 of intracellular water increases substantially just by being released from proteins (as in acute ischemia). Accordingly, experimental studies have suggested that the increase of free water and decrease of bound water fractions have a stronger impact on T2 than the overall water content [50].

**Figure 3:**
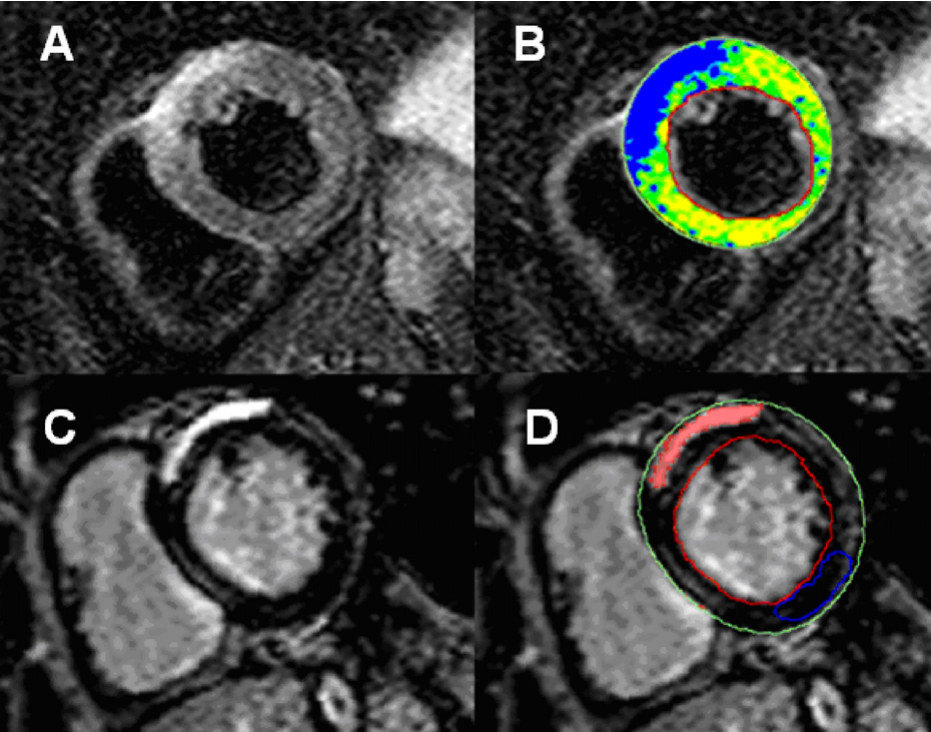
**Myocardial edema in a patient with active myocarditis**. **A**: T2-weighted CMR image showing subepicardial edema in the anteroseptal segment. **B**: Computer-aided signal intensity analysis of the T2-weighted image with color-coded display of relative signal intensity, normalized to skeletal muscle. Blue indicates a signal intensity ratio of myocardium/skeletal muscle of ≥2.0, indicating edema, green indicates normal signal intensity (1.4-1.9). **C**: Contrast-enhanced image (late gadolinium enhancement) showing a high signal intensity in the same region indicating necrosis. **D**: Computer-aided signal intensity analysis of the necrosis image with color-coded display of relative signal intensity, normalized to remote myocardium. Red indicates a signal intensity of >5 standard deviations above remote myocardium.

**Figure 4:**
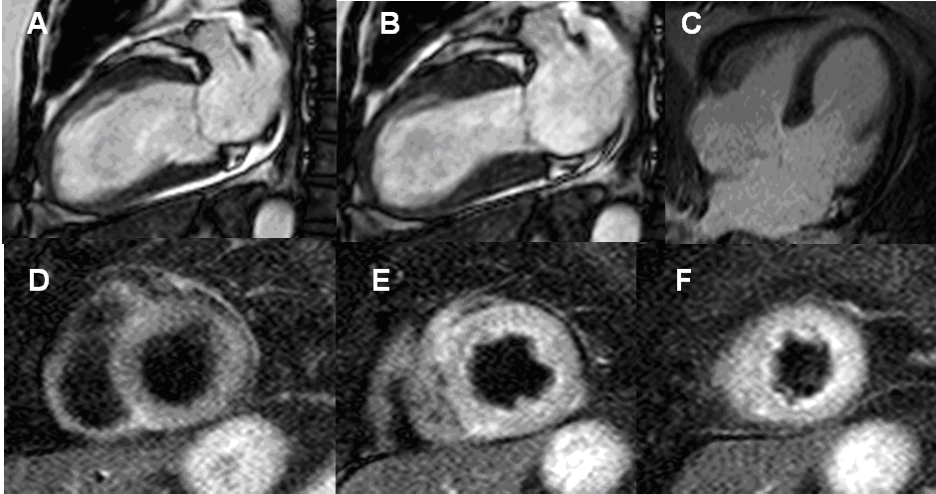
CMR Findings in a Patient With Stress-Induced Cardiomyopathy Takotsubo. There is characteristic apical contractile dysfunction (**A**, **B**) in the absence of late gadolinium enhancement (**C**).  T2-weighted images showing normal signal intensity of the basal myocardium (**D**), but global edema in the apical and midventricular myocardium matching the distribution of the LV wall motion abnormalities (**E**, **F**).

**Figure 5:**
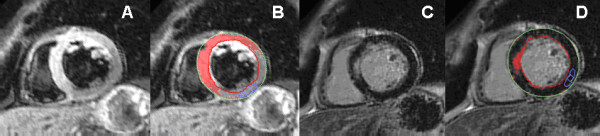
**Assessment of myocardial salvage after acute, reperfused myocardial infarction**. **A**: T2-weighted CMR showing high signal intensity of the anterior, anteroseptal and inferoseptal segments (area at risk). **B**: Computer-aided signal intensity analysis of the T2-weighted image normalized to normal, uninjured myocardium. Red indicates a signal intensity of >2 standard deviations above remote, uninjured myocardium. **C**: Contrast-enhanced image (late gadolinium enhancement) showing high signal intensity reflecting increased contrast accumulation in necrotic myocardium. **D**: Computer-aided signal intensity analysis of the late gadolinium enhancement image with color-coded display of relative signal intensity, normalized to remote myocardium. Red indicates a signal intensity of >5 standard deviations above remote, uninjured myocardium. The comparison of edema (panels A, B) with necrosis (panels C, D) shows myocardial edema in areas without necrosis, indicating major myocardial salvage.

**Figure 6:**
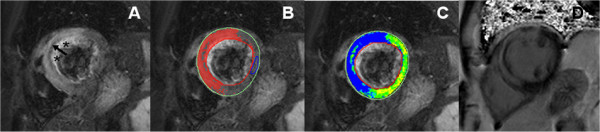
**T2 weighted imaging for detection of intramyocardial hemorrhage**. **A**: T2 images showing a hypointense core indicating intramyocardial hemorrhage within the area of myocardial edema (arrow). Asterisks refer to inadequately suppressed blood signal ("slow flow artefact") **B**: Computer aided signal intensity analysis normalized to normal myocardium and to skeletal muscle **(C)**. Contrast-enhanced image showing a transmural necrosis with a core of late microvascular obstruction (arrow) **(D)**.

Extensive preclinical and human studies have confirmed a close correlation between T2 and edema not only in (irreversible) myocardial infarction, but also in severe transient myocardial ischemia. Elevated myocardial T2 is also known to accompany myocarditis/inflammation [16,17], stress (Takotsubo) cardiomyopathy [18-20] and/or cardiac transplant rejection [14,15]. Notably, similar to LGE, the global, patchy, or subepicardial regional distribution patterns of edema in these entities typically are distinct from ischemic injury, which predominantly affects the subendocardium or is regionally transmural in a coronary artery territory (Figure 3, 4, 5 and 6).

### T2-weighted Sequences for Edema Assessment

Standard T2-weighted imaging of myocardial edema typically utilizes turbo spin-echo (TSE) readouts with or without fat saturation pulses, mostly combined with dark-blood preparation [51]. Currently, most of the clinical experience in visualizing myocardial edema has been reported for short-TI triple-inversion recovery prepared fast spin echo sequences (STIR). The inversion pulses for fat and blood suppression provide excellent contrast between regional edema and normal myocardium due to the dual suppression of the fat and flowing blood signal [52]. Furthermore, the inverse T1 weighting properties of these sequences increase their sensitivity to free tissue water [50]. Alternatively, double inversion recovery sequences, which may provide a higher SNR, have been used in clinical studies [8,53].

More recently, two new T2-weighted, SSFP-based sequences for cardiac application have been published, either using T2 preparation [54] or a hybrid approach with a spin echo pulse [55]. Both may offer a more robust image quality with preserved contrast-to-noise ratio when compared with STIR. Non-prepared SSFP sequences have been also used to detect myocardial edema with a moderate sensitivity and high specificity [56].

An alternative approach to T2-weighted imaging using signal intensity as a surrogate for T2 prolongation, is the direct determination of myocardial T2 relaxation times [57]. Thereby, several limitations associated with conventional T2-weighted imaging (see below) can be addressed, resulting in a potentially more reliable method for detection of myocardial edema. Furthermore this approach would overcome problems with identifying and verifying global edema and associated changes of myocardial T2. Clinical data, however, are still lacking.

### Limitations of T2-weighted CMR

The STIR technique produces useful images in most clinical cases, but can fail in some instances [58]. The inherently low signal-to-noise ratio and the relatively small differences in contrast-to-noise ratios between injured and normal myocardium remain challenging [46]. Therefore, a slice thickness of at least 8-10 mm is recommended to increase SNR. Furthermore, it is essential to have a uniform signal reception within the field of view; therefore, a reliable surface-coil intensity correction or the use of a body coil is important. The dark-blood preparation used in TSE T2-weighted imaging may introduce significant signal loss due to through-plane cardiac motion, typically most noticeable in the posterior wall. Such a minor signal loss of the inferior wall can be seen in Figure 5A. In some cases, such signal loss causes an intensity variation indistinguishable from the increase in T2 arising from edema, or it may cause normal myocardium to appear to have increased T2, resulting in a false-positive diagnosis [46,54,55]. The latter has been recently addressed by specialized techniques aiming to improve acquisition timing [59], which however, is difficult at higher heart rates. Particularly in patients with arrhythmia and other motion artifacts, such problems may result in non-diagnostic images.

Furthermore, an incomplete dark-blood preparation sometimes leaves a bright rim blood artifact adjacent to the endocardium ("slow flow artefact"), making it difficult to differentiate subendocardial edema from intracavitary blood (Figure 6A) [46]. One method to reduce this in clinical practice is to compare T2-weighted images of the same cardiac phase side-by-side with cine images to verify wall thickness [22].

Another limitation is the often qualitative nature of T2-weighted imaging. Interpretation depends on regional differences in myocardial signal intensity, which may vary depending on sequence parameters (echo times, slice thickness etc.). Especially when comparing edema with scars, cut-off values for defining abnormal vs. normal tissue for automated quantification are not sufficiently standardized between methods.

As mentioned above, two recently introduced techniques address several of these limitations, but these new methods are also qualitative and still depend on subjective interpretation of T2-weighted images and relative regional differences in myocardial signal intensity. Particularly, direct measurement of T2 by mapping techniques could overcome such limitations [57]. Furthermore, new sequences using shorter acquisition protocols, such as single-shot techniques [56], may provide a more robust image quality.

In summary, CMR protocols for edema detection still require further optimization. Despite these challenges, however, T2-weighted CMR usually provides images with diagnostic quality, allowing a clinically useful interpretation in most patients.

## Clinical Application of T2-Weighted CMR in Acute Cardiac Disease

### T2-weighted CMR in Acute Coronary Syndrome

CMR imaging is emerging as a diagnostic tool for the detection, differential diagnosis, management and prognostication of patients with suspected or established ACS [6,8,19,60,61]. Particularly, T2 weighted imaging plays a pivotal role in patients with acute chest pain by identifying myocardium not irreversibly injured but at risk of further injury. Furthermore, in patients with suspected ischemia and a history of previous infarcts, T2-weighted imaging can reliably differentiate acute myocardial injury from chronic infarcts [7].

A recent prospective study demonstrated that T2-weighted CMR improves diagnostic accuracy when characterizing patients with possible ACS who presented to the emergency department with chest pain. Adding T2-weighted imaging to a standard CMR protocol (function, perfusion, and scar imaging) increased the specificity, positive predictive value, and overall accuracy for detection of an ACS from 84% to 96%, 55% to 85%, and 84% to 93%, respectively [8]. Moreover, including T2-weighted imaging in the CMR protocol added significant value over clinical assessment and traditional cardiac risk factors.

T2-weighted imaging provides incremental diagnostic information above and beyond viability assessment, with acuity of cardiac disease being one of the most important determinants for patient management. Specifically, T2-weighted images can determine the acuity of many patients with unstable angina as early as 30 min after the onset of ischemia, a feature that cannot be ascertained by current generation biomarkers or CMR scar imaging using LGE [62].

A recent study extended this work and demonstrated that CMR with edema imaging has great potential to stratify patients admitted with Non-ST-Segment Elevation (NSTE) ACS by identifying higher-risk patients who would qualify for early invasive management strategies [9]. These findings indicate that detecting myocardial edema *in vivo *in patients with NSTE-ACS can be a powerful tool with major clinical implications. However, further randomized studies are warranted to study the impact of T2-weighted CMR imaging on selection of management strategies and ultimately on prognosis in patients with NSTE-ACS.

Edema imaging is also useful in patients with troponin-positive chest-pain and unobstructed coronary arteries. In this diagnostic challenging and important group of patients, T2-weighted imaging is complementary to LGE imaging for identifying the underlying etiology. The differential diagnosis includes ischemic infarction, myocarditis, stress-induced cardiomyopathy (Takotsubo) and/or other cardiomyopathies (e.g. dilated cardiomyopathy) [6,19,63]. In patients with infarction, edema will be transmural and localized in a single coronary territory with or without (aborted infarction) necrosis as defined by LGE [64] (Figure 5, 6). In inflammation/myocarditis, however, the regional distribution of edema is usually not reflective of a coronary perfusion bed and typically appears with a global or mainly subepicardial distribution [17] (Figure 3). The pattern of edema in Takotsubo cardiomyopathy is characterized by a global apical and midventricular edema matching the distribution of LV dysfunction in the absence or, rarely, presence of subtle focal or patchy myocardial scarring [18,19] (Figure 4). The exact pathophysiological mechanisms underlying the development of myocardial edema in Takotsubo cardiomyopathy remains unclear but inflammation, increased LV wall stress and/or transient ischemia appear pivotal. Importantly, edema imaging is not only helpful in establishing a diagnosis, but can also provide valuable insights into the underlying pathophysiology.

### T2-weighted CMR in Acute ST-elevation Myocardial Infarction

Experimental studies [10] have demonstrated that regional hyperintense areas on *in vivo *T2-weighted images obtained 2 days following left anterior descending coronary occlusion accurately define the ischemic area at risk in both reperfused [10] and nonreperfused infarctions [11]. The size of the edematous region reflects the perfusion bed of a coronary artery, which represents the area at risk. Thus, in a clinical setting of reperfused myocardial infarction, edema as defined by high T2 signal intensity is transmural, even if the acute irreversible injury (necrosis) is not.

Friedrich et al. [12] applied this technique to patients and systematically assessed myocardial salvage by comparing T2-weighted (area at risk) with LGE (infarct size) CMR images in 92 patients with reperfused infarction. In this study, the area at risk identified by T2 imaging were consistently transmural and exceeded areas of irreversible injury defined by LGE by 16 ± 11%. The salvaged area at risk remains LGE negative at follow-up as a result of successful reperfusion [65-68].

Furthermore, trials demonstrated that myocardial salvage assessment by CMR is a reproducible tool [69] that identifies and quantifies myocardial salvage in excellent agreement with SPECT [68,70] and angiographic scores of myocardial salvage [67,71]. These data clearly show that edema imaging enables the use of myocardial salvage as a robust marker for assessing the success of coronary revascularization in clinical settings.

While myocardial salvage assessed by CMR is independently associated with adverse LV remodelling [65] and more importantly with hard clinical endpoints (mortality and major adverse cardiac events) [13], quantifying the extent of the salvaged area at risk after revascularization might serve as a strong endpoint for clinical trials investigating novel reperfusion strategies.

Theoretically, there are advantages of measuring the salvaged area at risk over infarct size as an indicator of therapeutic efficacy in clinical trials. It is important to keep in mind that the size of the area at risk and the infarction depend on the size of the perfusion bed. Consequently, these parameters are subject to significant baseline differences between patients and even individual clinical events, independent of other clinical circumstances including revascularization. In clinical trials, this bias may account for more than 50% of infarct size variability [72,73]. As a more robust surrogate endpoint for clinical research, the myocardial salvage index is calculated by adjusting the volume of myocardial salvage to that of the area at risk and has been already successfully applied in clinical trials [74,75].

Recent studies have also demonstrated the potential of T2-weighted imaging to detect intramyocardial hemorrhage (IMH), a marker of severe reperfusion injury [76-79]. As IMH occurs only in acutely reperfused, infarcted myocardium, T2 values in these regions are determined by the relative contribution of two opposing mechanisms: 1) increase in T2 caused by tissue edema; 2) a drop in T2 induced by the paramagnetic effect of deoxyhemoglobin or degradation products of hemoglobin as found in hemorrhage or thrombus [80]. The resulting hypointense core within the area of tissue edema seen on T2-weighted images has been shown to be an independent predictor of adverse LV remodeling regardless of the initial infarct size and microvascular obstruction [76]. However, histological proof of the specificity of hypointense cores in T2-weighted images (vs. more specific T2*-weighted CMR) is still awaited [81] and the clinical significance of such findings have not been established yet. However, currently a multiparametric CMR protocol including T2-weighted imaging and late enhancement imaging for assessment of severe reperfusion injury is recommended.

## Alternative CMR Methods for Area at Risk Measurement

Recently also other CMR methods for area at risk measurement have been suggested. One of these methods is the endocardial extent of infarction as assessed by LGE CMR [82]. The pathophysiological background for this method is based on experimental studies showing that the endocardial extent of infarction is established approximately 40 minutes after coronary occlusion [83]. Thereafter, the infarcted area will increase by transmural progression from the endocardium to the epicardium, referred to as the wavefront phenomenon [83]. Thus, timely reperfusion is thought to limit the transmural infarct progression rather than the endocardial extent of infarction, implying that the endocardial extent of infarction could potentially be used for assessing the area at risk. However, in the situation of early reperfusion, infarction might be completely aborted [64,84] resulting in difficulties when assessing the area at risk based on infarct characteristics [85]. Consequently a recent study has demonstrated that the endocardial extent of infarction as assessed by LGE CMR underestimates the area at risk in comparison to T2-weighted imaging, especially in patients with early reperfusion and aborted myocardial infarction [85].

Another potential CMR method for area at risk measurement is to assess the dysfunctional myocardium in cine SSFP imaging [86]. Myocardial salvage can be defined using this approach as dysfunctional (cine imaging) but viable (no LGE) myocardium. Such a simple algorithm for myocardial salvage assessment is appealing, as measurement of the volume of the dysfunctional infarcted myocardium is simple, and the SSFP sequence for assessment of LV function is a robust and well-established CMR technique. However, such an approach is mainly limited by the fact that the complete area at risk exhibits functional impairment only at the time of coronary artery occlusion, whereas the extent of myocardial stunning may subside over time after reperfusion [87]. Consequently, T2-weighted CMR imaging early after infarction appears much more accurate and useful for quantifying myocardial salvage, since it can be derived up to one week after acute infarction.

Limiting the utility of a functional definition of the area at risk, there is evidence that areas of functional impairment have been found to be distinctly smaller than the area at risk as derived from T2-weighted imaging [12,13,65]; thus, the mere functional assessment likely underestimates the area at risk, especially in patients with early reperfusion and aborted myocardial infarction. This may in part be due to passive motion of the border zones, but also because of only minor impairment of function in less severely injury parts of the salvaged area at risk. Consequently, it was shown that patients with extensive myocardial salvage usually develop only minor regional wall motion abnormalities, whereas a large area of edema/area at risk can be detected [64]. Furthermore, the lack of a distinct border between normal and abnormal function makes it difficult to quantify the area and would allow for significant observer bias. Finally, clinical data using a functional area at risk measurement are limited/lacking, whereas T2-weighted imaging has been validated against histological [5] and angiographic measurements [67,71] of myocardium at risk and has been successfully applied in clinical trials [74,75].

Taken together other CMR methods for myocardium at risk assessment have significant limitations. Therefore T2-weighted CMR imaging currently seems to be the most accurate and best validated CMR technique for area at risk measurement.

## Future Challenges

Although T2-weighted CMR technology has improved substantially in recent years, it will further benefit from more robust protocols. T2 quantification (mapping) offers the potential for improved detection of myocardial edema, but further studies are warranted to evaluate the clinical applicability of this technique in the range of conditions that are known to globally or regionally affect the myocardial water status. Furthermore, scanning protocols have to be simplified and consolidated, and post-CMR processing and evaluation procedures need to be standardized and less time-consuming [6].

Future studies should address how much of a myocardial injury is needed to become apparent on CMR images of myocardial edema and how clinical and biological factors (e.g. intensity and/or duration of ischemia) affect the magnitude, duration, and time course of myocardial T2 changes. Furthermore, experimental studies are needed to clarify the relative importance of factors capable of influencing T2 signals during and after ischemia [88].

Studies in large, well-defined subsets of patients will be needed to define the additive value of T2-weighted imaging in specific clinical settings. For example it is unknown if cardioprotective strategies specifically targeting edema would help as adjunctive therapies to improve outcomes. Finally, as for all imaging techniques, there is the need for randomized studies comparing CMR-guided treatment decisions including edema imaging versus standard treatment to better understand how CMR and T2-weighted imaging can improve treatment and outcome in patients with ACS.

## Summary and Conclusions

Imaging myocardial edema with CMR in patients with acute cardiac disease provides useful incremental information on the acuity of myocardial injury, be it ischemic or non-ischemic.

In institutions with rapid access to CMR, the assessment of myocardial edema as an *in vivo *tissue marker for acute myocardial injury also significantly improves the clinician`s ability to stratify the risk in patients with acute chest pain syndromes and triage them to appropriate treatment.

Comprehensive CMR scans also allows for obtaining accurate information on LV function, size, morphology, perfusion, and scarring during the same scan (typically within 30 to 45 minutes), so has to be considered very efficient. Importantly, in combination with scar imaging, T2-weighted CMR of myocardial edema differentiates reversible from irreversible injury and can quantify myocardial salvage after coronary revascularization, with important implications for patient management and prognosis. Furthermore, it provides a very powerful, safe and cost-efficient endpoint for clinical trials on myocardial revascularization.

In conclusion, T2-weighted CMR is a validated, unique technique which should be considered as an essential diagnostic tool as part of a comprehensive CMR scan.

## Competing interests

The authors declare that they have no competing interests.

## Authors' contributions

Each author participated in researching the relevant articles and drafting and revising the manuscript.
